# Isolation of Phenolic Derivatives and Essential Oil Analysis of *Prangos ferulacea* (L.) Lindl. Aerial Parts

**Published:** 2017

**Authors:** Mohammad-Reza Delnavazi, Maryam Soleimani, Abbas Hadjiakhoondi, Narguess Yass

**Affiliations:** *Department of Pharmacognosy, Faculty of Pharmacy and Medicinal Plant Research Center, Tehran University of Medical Sciences, Tehran, Iran.*

**Keywords:** *Prangos ferulacea*, Apiaceae, coumarin, flavonoid, essential oil

## Abstract

*Prangos ferulacea* (L.) Lindl. (Apiaceae) is a medicinal plant distributed in Mediterranean regions, Caucasia and southwest of Asia. In the present study phytochemical constituents of the extract obtained from the aerial parts of *P. ferulacea* were investigated using various chromatographic and spectroscopic methods. Essential oil of the plant aerial parts was also analyzed using GC-MS. Five phenolic derivatives, isoimperatorin (1), ferudenol (2), caffeic acid glucosyl ester (3), isorhamnetin-3-O-β-D-glucopyranoside (4) and quercetin-3-O-β-D-glucopyranoside (5) were isolated from the aerial parts of *P. ferulacea* and their structures were elucidated using ^1^H-NMR, ^13^C-NMR, EI-MS and UV spectral analyses. Twenty-seven compounds were also identified in the essential oil of plant aerial parts, of which β-pinene (43.1%), α-pinene (22.1%) and -δ3-carene (16.9%) were characterized as main compounds. The results of this study introduce *P. ferulacea* as a source of potentially bioactive phenolic compounds and suggest it as an appropriate candidate for further studies.

## Introduction

The genus *Prangos* Lindl. from Apiaceae family, consists of about 30 species, mainly distributed in Irano-Turanian phytogeographic region (1). In flora of Iran, this genus is represented by 14 species including *Prangos ferulacea* (L.) Lindl. (2).

*P. ferulacea* is a perennial plant with up to 1.5 m height which grows in Eastern Europe, Turkey, Caucasia and southwestern Asia (2). This species is known as "Jâshir" in Iran and its aerial parts are used traditionally as laxative and against ruminant parasites (3, 4). In eastern Anatolia, the aromatic aerial parts of *P. ferulacea* are used as a flavor in cheese and its stem is also used as digestive, antidiabetic and antihypertensive agent (5, 6). Beside, *P. ferulacea* is used as animal fodder because of its valuable nutritive properties (7).

Literature review revealed that some biological properties such as antioxidant (5, 8), antibacterial (9, 10), antispasmodic (11), abortifacient (12), analgesic (13) and hepatoprotective (14) activities have been reported for the aerial parts of *P. ferulacea*. It has also been reported that the roots of this medicinal species improve serum glucose and lipid profile in diabetic rats (15). 

So far, several phytochemical studies have been conducted on essential oils and extracts obtained from the different parts of *P. ferulacea* (16-28). Tawaha *et al*. (2001) reported the isolation of four coumarins, herniarin, umbelliferone, scopoletin, and osthenol, along with two furanocoumarins, xanthotoxin and imperatorin from the aerial parts of *P. ferulacea* (23). A number of coumarin and furanocoumarin derivatives have also been reported from the roots of this species (24-28).

In the present study, we report essential oil constituents and isolation of the five phenolic derivatives, isoimperatorin (1), ferudenol (2), caffeic acid glucosyl ester (3), isorhamnetin-3-O-β-D-glucopyranoside (4) and quercetin-3-O-β-D-glucopyranoside (5) from the aerial parts of *P. ferulacea* growing in Northwest of Iran. To the best of our knowledge, this is the first report on isolation and structure elucidation of these phenolic derivatives from the *P. ferulacea* aerial parts.

## Experimental

*General procedures*

^1^H-NMR and ^13^C-NMR spectra were acquired in CDCl_3_ and DMSO-*d*_6_ on a Bruker Avance DRX 400 spectrometer. EI-MS spectra were obtained on a Hewlett-Packard model 5973 HP system. UV spectra were recorded in methanol (and after the addition of shift reagents) on a CECIL 7250 spectrophotometer. 

Silica gel (230-400 mesh, Merck), fully endcappedRP-18 (230-400 mesh, Fluka) and Sephadex LH-20 (Fluka) were applied for column chromatographies. Preparative thin layer chromatography (PTLC) was also performed on handmade silica gel 60 GF_254_ (Merck) plates. Pre-coated silica gel GF_254_ aluminum sheets (Merck) were used for TLC and monitoring of the spots were carried out under UV (254 and 366 nm) and by spraying with anisaldehyde-H_2_SO_4_ reagent followed by heating at 120°C for 5 min. All of the solvents were also obtained from Merck chemical company.

*Plant material*

The flowering aerial parts of *P. ferulacea* were collected from the Razi border, located in Khoy County (West-Azerbaijan Province, Northwest of Iran) in June 2014. The plant was identified by botanist Dr. Yousef Ajani, Institute of Medicinal Plants, Academic Center for Education, Culture and Research (ACECR), Karaj, Iran.

*Extraction and fractionation*

The air-dried aerial parts of *P. ferulacea* (1kg) were grinded and extracted exhaustively with MeOH by maceration method at the room temperature (10×12 L and 48 h each time). The combined extracts were concentrated by a rotary evaporator at 45°C. The obtained total extract (274.58 g) was fractionated successively with petroleum ether and chloroform using liquid-liquid extraction, to get three main fractions, petroleum ether, chloroform and methanol (residue) fractions.

*Isolation and purification of compounds*

The petroleum ether fraction (12 g) was dissolved in petroleum ether (200 ml) and transferred into a flask. Following the addition of ethanol (30 ml) to above solution, a white precipitate was appeared which was then separated by filtration and named fraction E2.

The fraction E2 (7 g) was moved over a silica gel column (4.5×30 cm) and eluted with *n*-hexane-EtOAc (9:1 to 5:5) to get fourteen fractions (E2A-E2N). Fraction E2B (52 mg) was chromatographed on silica gel PTLC with *n*-hexane-EtOAc (8:2), to give compound 1 (9 mg). Compound 2 (5 mg) was also obtained from the fraction E2I (18 mg) by chromatography on silica gel PTLC (*n*-hexane-EtOAc, 6:4). A part of methanol fraction (6 g) was moved in two divided portions over a sephadex LH-20 column (4×25 cm) and eluted with MeOH-H_2_O (8:2), to get three fractions (M1-M3). Reversed phase C18 column chromatography (3×15 cm) of the fraction M2 (2 g) with ACN (acetonitrile)-H_2_O (1:9-3:7) yielded eleven fractions (M2A-M2K). Chromatography of the fraction M2A (250 mg) on a RP-18 column (1.5×20 cm) using ACN-H_2_O (0.5:9.5) resulted in isolation of compound 3 (18 mg). Fraction M2H (38 mg) was rechromatographed on a RP-18 column (1×20 cm) with ACN-H_2_O (1:9-2:8) to obtain compound 4 (21 mg). Compound 5 (7 mg) was also achieved from the fraction M2I (34 mg) through RP-18 column chromatography (1×20 cm) using ACN-H_2_O (1.5:8.5) as eluent and its impurities were removed over a sephadex LH-20 column (MeOH-H_2_O, 8:2).

*Isolation of essential oil*

Essential oil of the plant aerial parts was extracted using hydro-distillation for 4 h by a Clevenger-type apparatus. The prepared oil was subsequently dried over anhydrous sodium sulfate and stored at 4°C in the dark until analysis.

*GC-MS analysis*

An Agilent 6890 gas chromatograph (Column: BPX5, 30 m × 0.25 mm (id), 0.25 µm film thickness) equipped with a MS detector (Agilent 5973, EI mode at 70 eV, 220 °C) was applied for the essential oil analysis. The flow rate of carrier gas (Helium) was 0.5 ml min^-1^. The oven temperature was raised from 50 °C to 240 °C at a rate of 3 °C per minute and then raised to 300 °C at a rate of 15 °C and finally maintained at 300 °C for 3 min. The injection temperature was 290 °C, and the oil sample (1.0 µL) was injected with a split ratio of 1:30. The Kovats retention indices (KI) of the compounds were calculated using a homologous series of *n*-alkanes (C_8_-C_30_) injected in conditions equal to the samples. Identification of the chemical constituents was performed using Wiley7n.l online library, as well as by direct comparison of their MS spectra and KIs with data published in the literature for standard compounds (29).

Results

**Figure 1:**
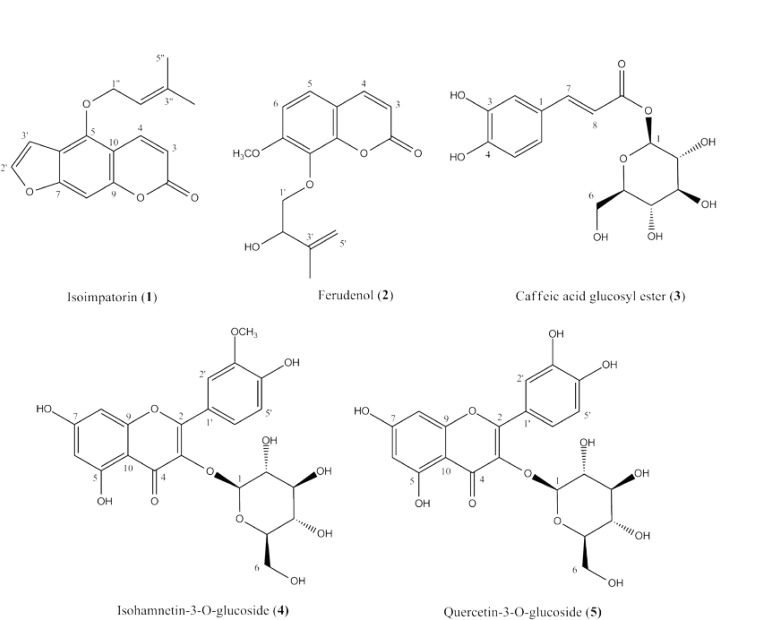
The structures of isolated compounds (1-5) from the aerial parts of *P. ferulacea*

Phytochemical investigation of the aerial parts of *P. ferulacea* yielded the isolation of five compounds. The structures of isolated compounds were established as isoimperatorin (1), ferudenol (2), caffeic acid glucosyl ester (3), isorhamnetin-3-O-β-D-glucopyranoside (4) and quercetin-3-O-β-D-glucopyranoside (isoquercetin) (5) (Figure 1) using their ^1^H- & ^13^C-NMR, EI-MS and UV spectral analyses, and also by comparison with related data published in the literature (27, 30-33). 

*Spectral data of the isolated compounds*

*Compound *1; *Isoimperatorin*: White crystals, ^1^H-NMR (CDCl_3_, 400 MHz): δ 8.17 (1H, *d*, *J*= 9.8 Hz, H4), 7.60 (1H, *d*, *J*= 2.2 Hz, H2'), 7.15 (1H, *s*,H8), 6.97 (1H, *d*, *J*= 2.2 Hz, H3'), 6.26 (1H, *d*, *J*= 9.8 Hz, H3), 5.55 (1H, *t*, *J*= 6.8 Hz, H2''), 4.93 (2H, *d*, *J*= 6.8, H1''), 1.81 (3H, *s*, H5''), 1.71 (3H, *s*, H4''); ^13^C-NMR (CDCl_3_, 100 MHz); 161.1 (C2), 158.2 (C7), 152.6 (C9), 148.8 (C5), 144.8 (C2'), 139.7 (C3''), 139.4 (C4), 119.2 (C2''), 114.9 (C6), 112.3 (C3), 107.3 (C10), 105.1 (C3'), 95.1 (C8), 69.8 (C1''), 25.8 (C5''), 18.3 (C4''); UV (MeOH) λ_ max_: 249, 260, 309 (30).

*Compound* 2; *Ferudenol*: White crystals, ^1^H-NMR (CDCl_3_, 400 MHz): δ 8.52 (1H, *s*, OH), 7.65 (1H, *d*, *J*= 9.6 Hz, H4), 7.37 (1H, *d*, *J*= 8.6 Hz, H5), 6.88 (1H, *d*, *J*= 8.6 Hz, H6), 6.27 (1H, *d*, *J*= 9.6 Hz, H3), 4.89 (2H, *d*, *J*= 21.0 Hz, H5'), 4.62 (1H, *dd*, *J*= 8.0, 5.5 Hz, H2'), 3.96 (3H, *s*, OCH_3_), 3.28 (1H, *dd*, *J*= 11.3, 8.0Hz, H1'a), 3.17 (1H, *dd*, *J*= 11.3, 5.5 Hz, H1'b),1.92 (3H, *s*, H4'); UV (MeOH) λ_ max_: 210, 253 (sh), 322.; EI-MS (m/z): 276 [M^+^], 258, 219, 203, 189, 175, 131 (27).

*Compound* 3; *Caffeic acid glucosyl ester*: Light brown solid, ^1^H-NMR (DMSO-*d*_6_, 400 MHz): δ 7.46 (1H, *d*, *J*= 15.3 Hz, H7), 7.04 (1H, *brs*, H2), 6.95 (1H, *brd*, *J*= 8.1, H6), 6.75 (1H, *d*, *J*= 8.1, H5), 6.23 (1H, *d*, *J*= 15.3 Hz, H8), 4.10 (1H, *d*, *J*= 7.6 Hz, H1'), 2.8-3.8 (6H, H2'-6'); ^13^C-NMR (DMSO-*d*_6_, 100 MHz): δ 170.1 (C9), 150.2 (C4), 149.6 (C3), 146.3 (C7), 128.3 (C1), 125.1 (C6), 118.4 (C8), 117.5 (C5), 115.1 (C2), 102.4 (C1'), 79.1 (C3'), 74.6 (C2', C5'), 69.1 (C4'), 60.7 (C6'); UV (MeOH) λ_ max_: 206, 245 (sh), 300 (sh), 328., +AlCl_3_: 206, 259, 304 (sh), 366., +AlCl_3_/HCl: 206, 272 (sh), 302 (sh), 342 (31).

*Compound* 4; *Isorhamnetin-3-O-β-D-glucopyranoside*: Yellow powder,^ 1^H-NMR (DMSO-*d*_6_, 400 MHz): δ 8.06 (1H, *brs*, H2'), 7.40 (1H, *brd*, *J*= 8.5 Hz, H6'), 6.91 (1H, *d*, *J*= 8.5 Hz, H5'), 6.29 (1H, *brs*, H8), 6.09 (1H, *brs*, H6), 5.50 (1H, *d*, *J*= 6.8 Hz, H1''), 3.82 (3H, *s*, OCH_3_), 3.0-4.0 (6H, H2''-6''); ^13^C-NMR (DMSO-*d*_6_, 100 MHz): δ 177.0 (C4), 167.3 (C7), 161.1 (C5), 156.6 (C2), 155.2 (C9), 149.8 (C3'), 145.9 (C4'), 133.4 (C3), 122.8 (C6'), 121.2 (C1'), 115.6 (C5'), 111.3 (C2'), 103.6 (C10), 101.0 (C1''), 99.5 (C6), 93.9 (C8), 77.5 (C3''), 76.5 (C5''), 74.1 (C2''), 69.9 (C4''), 60.9 (C6''), 55.6 (OCH_3_); UV (MeOH) λ_ max_: 253, 335, 360 (sh)., +AlCl_3_: 365, 266., +AlCl_3_/HCl: 266, 305 (sh), 355, 404 (sh)., +NaOMe: 272, 329 (sh), 395., +NaOAc: 332, 269; EI-MS (m/z): 299 [Aglycon^+^], 149, 137 (32).

*Compound* 5; *Quercetin-3-O-β-D-glucopyranoside (Isoquercetin)*: Yellow powder, ^1^H-NMR (DMSO-*d*_6_, 400 MHz): δ 8.28 (1H, *brs*, H2'); 7.35 (1H, *brd*, *J*= 7.9 Hz, H6'); 6.82 (1H, *d*, *J*= 8.3 Hz, H5'); 6.40 (1H, *brs*, H8); 6.20 (1H, *brs*, H6); 5.23 (1H, *d*, *J*= 5.9 Hz, H1'') 3.0-4.0 (6H, H2''-6''); ^13^C-NMR (DMSO-*d*_6_, 100 MHz): δ 177.3 (C4), 164.5 (C7), 161.2 (C5), 156.3 (C2), 156.1 (C9), 148.5 (C4'), 144.8 (C3'), 133.2 (C3), 121.5 (C6'), 121.1 (C1'), 116.1 (C5'), 115.2 (C2'), 103.8 (C10), 100.8 (C1''), 98.7 (C6), 93.5 (C8), 77.5 (C5''), 76.4 (C3''), 74.0 (C2''), 69.9 (C4''), 60.9 (C6''); UV (MeOH) λ_max_: 259, 368., +AlCl_3_: 269, 304 (sh), 421., + NaOMe: 272, 411., +NaOAc: 262, 368 (33).

The hydrodistillation of the plant aerial parts yielded 0.2% (V/W) pale yellowish oils. Twenty seven compounds (99.25% of the total oil) were identified as a result of GC-MS analysis of *P. ferulacea *essential oil, among them β-pinene (43.1%), α-pinene (22.1%) and δ-3-carene (16.9%) were characterized as main compounds (Table 1). The results also indicated that monoterpene hydrocarbons (94.45%) were the main group of constituents in *P. ferulacea *essential oil.

**Table 1:** Chemical composition of the essential oil of *P. ferulacea* aerial parts

No.	Compounds^a^	KI^b^	%	No.	Compounds	KI	%
**1**	α-thujene	926	0.10	**18**	trans-verbenol	1142	0.50
**2**	α-pinene	938	22.1	**19**	pinocarvone	1163	0.36
**3**	camphene	947	0.13	**20**	*p*-cymene-8-ol	1183	0.67
**4**	sabinene	969	0.57	**21**	myrtenal	1194	0.23
**5**	β-pinene	975	43.13	**22**	β-elemene	1388	0.61
**6**	β-myrcene	989	1.92	**23**	β-caryophyllene	1415	0.29
**7**	δ-3-carene	1008	16.88	**24**	elemol	1548	0.14
**8**	α-terpinene	1016	0.12	**25**	caryophyllene oxide	1582	0.26
**9**	*p*-cymene	1021	0.06	**26**	α-bisabolol	1687	0.46
**10**	*o*-cymene	1023	0.95	**27**	osthole	2144	1.28
**11**	limonene	1024	2.06				
**12**	β-phellandrene	1027	1.89		Monoterpene hydrocarbons		94.45
**13**	(Z)-β-ocimene	1032	0.07		Oxygenated monoterpenes		1.76
**14**	(E)-β-ocimene	1046	0.10		Sesquiterpene hydrocarbons		0.90
**15**	γ-terpinene	1054	0.26		Oxygenated sesquiterpenes		0.86
**16**	α-terpinolene	1085	3.90		Non-terpenes		1.28
**17**	*p*,α-dimethylstyrene	1099	0.21		Total identified		99.25

a Identified compounds listed in order of elution from BPX5 column;

b Kovats retention indices to C_8_-C_30 _*n*-alkanes on BPX5 column.

## Discussion

Two coumarin derivatives, isoimperatorin (1) and ferudenol (2) were isolated from the petroleum ether fraction of *P. ferulacea* aerial parts. These compounds (1 and 2) have been previously reported from the roots of *P. ferulacea* (24, 27, 28). A phytochemical study on *P. uloptera* aerial parts, however, reported the isolation of five other coumarin derivatives, xanthotoxin, prangenin, scopoletin, deltoin and prangolarin from its *n*-hexane extract (34). In 2006, Pokharel *et al*. showed that isoimperatorin possesses a considerable hepatoprotective effects against aflatoxin B_1_ through induction of glutathione S-transferase α (GSTα) and direct inhibition of CYP1A activity (35). It has also been reported that this compound (1) exerts a potent anti-inflammatory activity via cyclooxygenase-2 and 5-lipoxygenase inhibitory activity, as well as by inhibition of TNF-α-stimulated VCAM-1 (vascular cell adhesion molecule-1) expression (36, 37). Furthermore, antibacterial effects of the isoimperatorin against *Mycobacterium tuberculosis* (38) and its acetylcholinesterase (AChE) inhibitory activity (39) have been demonstrated during previous biological investigations. Phytochemical study on the methanolic fraction of the plant aerial parts also yielded caffeic acid glucosyl ester (3), together with two flavonol glycosides, isorhamnetin-3-O-β-D-glucopyranoside (4) and quercetin-3-O-β-D-glucopyranoside (Isoquercetin) (5). Caffeic acid, a bioactive phenolic compound, is found in many plant families, alone or in combination with sugars, quinic acid (caffeoylquinic acids), phenyl ethanol (caffeic acid phenethyl esters) and etc. (40). A review of literature on caffeic acid derivatives revealed that the glucose could be connected to caffeic acid through OH-3, OH-4 or COOH groups (31, 41, 42). In the present study, the structure of caffeic acid glucosyl ester was confirmed for compound 3, based on the observed bathochromic sift (+38 nm), followed by addition of AlCl_3 _to the methanolic solution of compound 3 which was indicated to the presence of a *ortho* dihydoxy group in compound 3 (Figure 1) (43). Caffeic acid glucosyl ester has also been reported as 2-methyl-3-[3'-O-caffeic acid glucosyl ester]-γ-pyrone from the aerial parts of *P. tschimganica *(44)*. * Two isolated flavonoids, isorhamnetin-3-O-β-D-glucopyranoside (4) and quercetin-3-O-β-D-glucopyranoside (Isoquercetin) (5), have been documented for their antioxidant (45, 46), α-glucosidase inhibitory (45, 46), anti-inflammatory (47, 48), antimicrobial (49) and antidiabetic (50) activities. Isorhamnetin-3-О-β-D-glucopyranoside has also been reported as an antiobesity agent with antiadipogenic activity on 3T3-L1 adipocytes (51).

Essential oil analysis of the *P. ferulacea* aerial parts resulted to the identification of twenty seven compounds, mainly β-pinene (43.1%), α-pinene (22.1%), δ-3-carene (16.9%) (Table 1). Osthole, a pernylated coumarin was identified in the plant essential oil with the relative percentage of 1.28%. This compound has been previously reported from the roots of *P. ferulacea* with antispasmodic effects on ileum contraction (11). Previous reports on essential oil composition of the different parts of *P. ferulacea *have been summarized in Table 2. 

**Table 2:** The results of essential oil analysis of *P. ferulacea* from the previous and present studies

**Location of plant collection**	**Date**	**Method**	**Part(s)**	**Main compounds (%)**	**Reference**
Khoy (West-Azerbaijan, Iran)	June 2012	HD^a^	aerial part	β-pinene (43.1%) α-pinene (22.1%) δ-3-carene (16.9%)	Present study
Isfahan (Iran)	July 1996	SD^b^	aerial part	β-pinene (22.9%) δ-3-carene (16.0%) α-pinene (12.6%)	(16)
Myaneh (East-Azerbaijan, Iran)	July 2005	HD	aerial part	(E)-anethole (95.0%) α-pinene (1.2%)	(17)
Myaneh (East-Azerbaijan, Iran)	May 2005	HD	leaves & stems	α-pinene (57.0%) β-pinene (4.5%) (E)-anethole (3.9%)	(17)
Sirjan (Kerman, Iran)	June 2010	HD	leaves	α-pinene (28.2%) δ-3-carene (15.3%) limonene (8.1%)	(21)
Myaneh (East-Azerbaijan, Iran)	-	HD	flowers	α-pinene (42.2%) cis-ocimene (36.3%)	(18)
Tosya (Kastamonu, Turkey)	Sept. 1994	HD	fruits	γ-terpinene (30.2%) α-pinene (16.7%) p-cymene (9.8%)	(20)
Sanandaj (Kordestan, Iran)	July 2001	SD	fruits	β-pinene (33.0%) α-pinene (10.1%) δ-3-carene (10.0%)	(16)
Shemshak (Tehran, Iran)	June 2006	HD	fruits	chrysanthenyl acetate (26.5%) limonene (19.6%) α-pinene (19.5%)	(19)
Sirjan (Kerman, Iran)	June 2010	HD	fruits	α-pinene (25.4%) 3-n-butyl phthalide (13.8%) limonene (10.6%)	(21)
Myaneh (East-Azerbaijan, Iran)	-	HD	fruits	α-pinene (61.3%) cis-ocimene (9.7%)	(18)
Yasouj (Kohkiluye boyerahmad, Iran)	June 2010	HD	roots	δ-3-carene (22.5%) β-phellandrene (11.8%) α-pinene (8.6%)	(22)

A dash (-) indicate not reported.

a Hydrodistillation,

b Steam distillation.

Sefidkon *et al*. (1998) reported β-pinene (22.9%), δ-3-carene (16.0%) and α-pinene (12.6%) as the main compounds of the essential oil of *P. ferulacea* aerial parts from Isfahan (center of Iran) (16). The comparison of the results represented in this paper with the former study on *P. ferulacea* essential oil from Isfahan province (Iran) revealed that α-thujene, α-pinene, camphene, β-pinene, β-myrcene, δ-3-carene, α-terpinene, *p*-cymene, (Z)-β-ocimene, (E)-β-ocimene, γ-terpinene, β-caryophyllene and α-bisabolol are the compounds present in both essential oil samples (16). Furthermore, in another study on essential oil of the plant flowering aerial parts from East-Azerbaijan (northwest of Iran), (E)-anethole (95.0%) and α-pinene (1.2%) were characterized as its main compounds (17). (E)-anethole, an aromatic major compound of the last mentioned study, however, was not identified in our analysed oil sample (17). The present study reports the monoterpene hydrocarbons (94.5%) as main group of constituents in *P. ferulacea *essential oil, whereas two previous reports from Isfahan and East-Azerbaijan provinces (Iran) reported the level of monoterpene hydrocarbons at 64.0 and 2.7%, respectively (16, 17). Genetic variations and climatic conditions could be considered as main factors involved in the chemical differences in composition of the essential oils obtained from *P. ferulacea* aerial parts (52).

## Conclusion

The results of present study on isolation of bioactive phenolic derivatives (**1**-**5**) from the aerial parts of *P. ferulacea* emphasize the therapeutic potentials of this medicinal species and suggest it as an appropriate candidate for further biological and pharmacological research. This study also introduces *P. ferulacea* as a plant with monoterpene rich oil and report β-pinene (43.1%) as the main compound of its aerial parts oil. 
